# Maternal Distress/Coping and Children's Adaptive Behaviors During the COVID-19 Lockdown: Mediation Through Children's Emotional Experience

**DOI:** 10.3389/fpubh.2020.587833

**Published:** 2020-11-19

**Authors:** Serena Petrocchi, Annalisa Levante, Federica Bianco, Ilaria Castelli, Flavia Lecciso

**Affiliations:** ^1^Institute of Communication and Health, Università della Svizzera Italiana, Lugano, Switzerland; ^2^Department of History, Society, and Human Studies, University of Salento, Lecce, Italy; ^3^Lab of Applied Psychology, Department of History, Society, and Human Studies, University of Salento, Lecce, Italy; ^4^Department of Human and Social Sciences, University of Bergamo, Bergamo, Italy

**Keywords:** COVID-19, SARS-CoV-2, lockdown, quarantine, distress, coping, emotions, parent-child

## Abstract

The present study focused on the psychological impact that the lockdown due to coronavirus disease-19 (COVID-19) had on families in Italy. During the COVID-19 pandemic, the Italian government imposed a strict lockdown for all citizens. People were forced to stay at home, and the length of the lockdown was uncertain. Previous studies analyzed the impact of social distance measures on individuals' mental health, whereas few studies have examined the interplay between the adults' functioning, as parents, during this period and the association with the child's adjustment. The present study tested if maternal distress/coping predicts children's behaviors during the COVID-19 lockdown, hypothesizing a mediation effect *via* children's emotional experience. Participants were 144 mothers (*M*_*age*_ = 39.3, 25–52, *SD* = 5.6) with children aged 5–10 years (*M*_*age*_ = 7.54, *SD* = 1.6, 82 boys); mothers answered to an online survey. Results indicated that mothers with higher exposure to COVID-19 showed higher levels of distress and higher display of coping attitudes, even if in the structural equation modeling model, the COVID-19 exposure was not a predictor of mothers' distress. Compared with mothers with good coping skills, mothers with higher stress levels were more likely to attribute negative emotions to their children at the expense of their positive emotions. Moreover, children's emotions acted as mediators between maternal distress/coping and children's adaptive/maladaptive behaviors. In conclusion, it is important to support parents during pandemic emergence, by providing them with adequate information to manage the relationship with their children, to reduce their level of distress and to enhance their coping abilities.

## Introduction

In December 2019, a new coronavirus disease (COVID-19) emerged from Wuhan, China, spreading an epidemic of the severe acute respiratory syndrome coronavirus 2 (SARS-CoV-2) in most other countries, leading the World Health Organization to declare a global pandemic in March 2020. COVID-19 represents a challenge for citizens, health-care institutions, policy makers, and psychologists because it poses a serious threat to psychological and physical health (1). From December 2019 to April 2020, 928,437 worldwide cases of COVID-19 have been reported, including 46,891 deaths (2, 3). In Italy, in March and April, when the present research was administered, the official number of cases reached 110,574 (4); however, research suggests that the estimates are higher than those reported in the official counts (5). Similarly, there were 216,721 new cases in the U.S., 102,136 in Spain, 73,522 in Germany, 56,989 in France, and 56,989 in the U.K.

On March 8, the Italian government decided to lockdown citizens in Northern Italy, and on the subsequent day, the lockdown was extended to all the other regions, with almost 60 million people confined at home. The lockdown ended on May 4. The present study focuses on the psychological impact of the COVID-19 lockdown on families in Italy, when the country was the second most affected after China for the number of infections and deaths. Although the positive impact of quarantine and lockdown on the spread of an epidemic is well-established (6, 7), it is also well-known that these measures have a detrimental effect on an individual's mental health (8). Research conducted during previous pandemics (9, 10) and COVID-19 (11, 12) demonstrated that under quarantine conditions, depression, anxiety, anger, and confusion increased in adults. Studies on Italian citizens (13, 14) found that 5.1% of individuals reported post-traumatic stress disorder (PTSD) symptoms, and that 48.2% reported low psychological well-being. Regarding the latter variable, they found lower levels in women than in men and in individuals who knew people who were affected, who have had direct exposure or were uncertain about their exposure to COVID-19, and who did not know if they were infected. Similarly, Forte et al. (14) reported high levels of general psychopathological symptoms, anxiety, and PTSD symptoms in the general Italian population during the mandatory lockdown for COVID-19. Casagrande et al. (15) also found that the COVID-19 pandemic impacted on sleep disorders, sleep quality, and psychological distress in general.

Despite this growing evidence on the general psychological consequence of the COVID-19 lockdown, few studies have examined the interplay between adults' functioning, as parents, and their children's functioning. Research to date has considered the psychological impact of the COVID-19 pandemic on the general adult population. There is a gap in the knowledge regarding the impact of COVID-19 and its consequent lockdown on adults' functioning as parents. The present research intends to contribute in this direction by focusing on the relations between maternal distress/coping and children's behaviors during the COVID-19 lockdown, hypothesizing a mediation effect *via* children's emotional experience. During the lockdown, parents were challenged not only by distress due to the uncertainty of the future, health worries, and social isolation but also by the increased requests associated with their parental role (16, 17). The closure of schools and the lockdown requested a drastic change in children's management with the consequence that parents while taking care of children struggled to combine family life and work in smart modality or were unable to work (18, 19). Moreover, the increasing time spent with their children exposed parents to the likelihood of experiencing more than usual parenting hassles (i.e., child's complaining, picky eating, and quarrels among siblings) exacerbated by the challenging time. As a consequence, the gap between the requests due to the parental role and the necessary resources (both at the practical and at the emotional level) to accomplish them may have become larger during the lockdown, thus resulting in an increase in parental distress as defined by Abidin (20). Children, as well, were exposed to many difficulties during the lockdown. They experienced a breakdown in their daily routines, and they had no chance to discharge their physical energy and to meet their peers because of home confinement (21). Parents and children, therefore, may have experienced high distress during the Italian lockdown due to the COVID-19 pandemic.

Previous research on parents and children during quarantine due to SARS (22) or H1N1 (23) indeed reported an increase in emotional disturbance and exhaustion, low mood, and irritability. Recently, Di Giorgio et al. (23) reported worse sleep quality and distortion of time experience in Italian mothers and children during the lockdown, plus an amount of emotional symptoms and self-regulation difficulties in children. In this framework, it is likely that children's adaptive/maladaptive behaviors eventually assumed during the lockdown closely mirrored the family emotional context in which they lived (24, 25), as first empirical evidence shows (17). According to the tripartite model of the impact of the family on children's emotion regulation and adjustment, proposed by Morris et al. (26), parents affect children's emotional modality to answer to events through (i) modeling/imitation processes, (ii) how they respond to children's emotions, and (iii) the general affective environment in which the child is dived in at home. Specifically, during the lockdown, it is plausible that the emotions of fear, worries, and overall distress felt by the parents resonated in children through the mechanisms of contagion of affect induction described by Denham et al. (27). To be able to regulate these feelings and the general distress caused by circumstances, for the child, how his/her parents were dealing with the difficulties of the pandemic period has been probably crucial, leading them to know how to respond, think, or feel in this specific situation (27). At the same time, parental distress is known to interfere with parenting practices that, in turn, play a role in the regulation of children's behavior and emotions (28). When parents manage to be supportive with their children in stressful situations, their children are, in turn, less exposed to the risk of being emotionally over-aroused. Therefore, such children are in conditions to manage their behaviors in a virtuous circle (29, 30).

Many empirical data go in this direction. For example, high levels of parental distress have been shown to influence children's behavior (27, 31–33) and their vulnerability to both internalizing (34, 35) and externalizing problems (36, 37). Therefore, we expected to find the following:

Hypothesis 1—Maternal distress during the COVID-19 lockdown has affected children's adaptive behaviors through the mediation of children's negative and positive emotional reactions. Specifically, it was expected that parental distress, influenced by COVID-19 exposure, would be associated with the children's positive and negative emotions, with negative and positive signs, respectively, which, in turn, would be associated with a range of children's adaptive behaviors.

**Figure 1:**
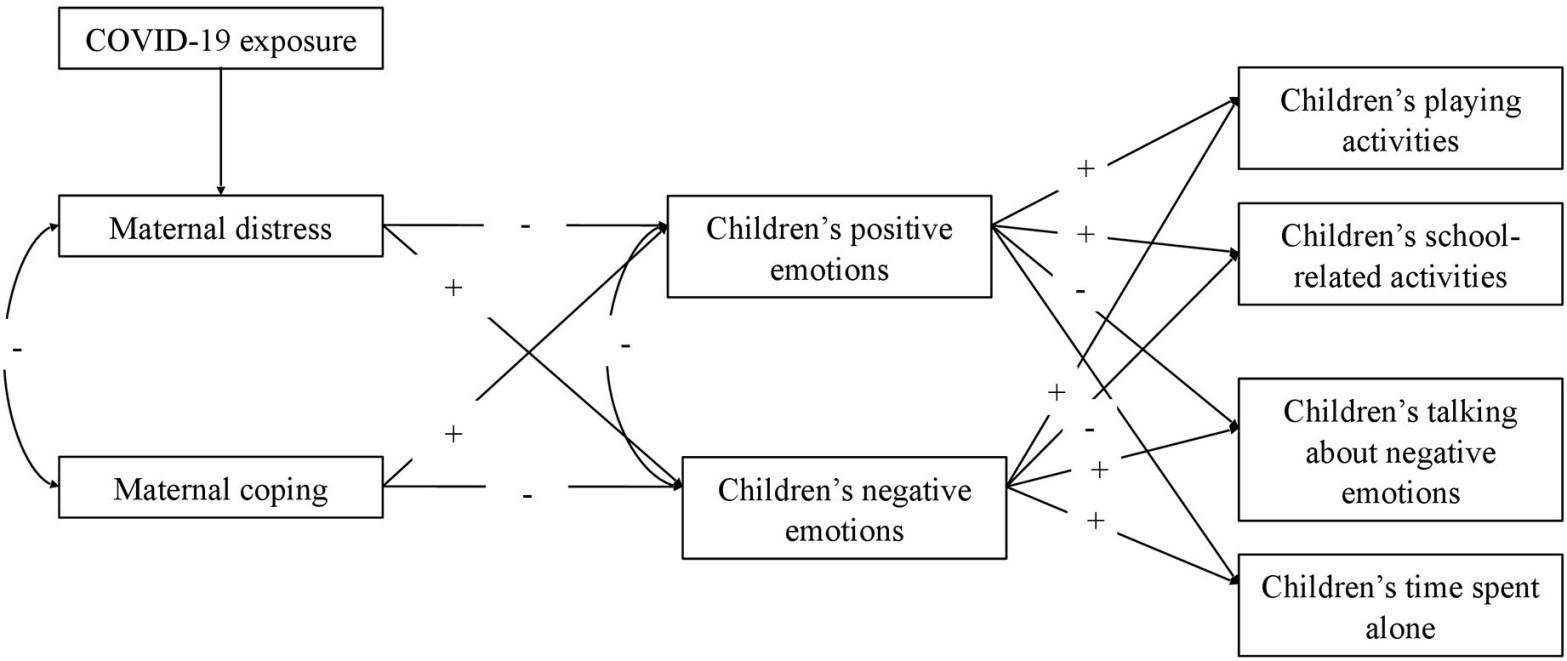
The hypothesized model.

The relevance of parental coping abilities (i.e., cognitive and behavioral efforts to manage difficult situations) for children's adaptive behaviors and emotional answers is evident in the rich literature linking parental approaches to children's negative emotions and children's ability to regulate emotions and behaviors [e.g., (38–40)]. Furthermore, clinical literature showed that children of depressed mothers utilized less effective strategies to deal with emotions than children of not depressed mothers (41, 42). Therefore, we expected to find the following:

Hypothesis 2—Maternal coping abilities during the COVID-19 lockdown have had an effect on children's adaptive behaviors through the mediation of children's negative and positive emotional reactions. Particularly, maternal coping abilities would be expected to influence children's positive and negative emotions, with positive and negative signs, respectively, which, in turn, would be linked to a range of children's behavioral activities. Figure 1 shows the full model tested.

## Methods

### Procedure

Data were collected by an online mother-reported survey developed on *Qualtrics XM* and widespread through the main social media platforms (i.e., WhatsApp and Facebook). Data were collected from April 1 to May 4 2020, while families were forced at home under the lockdown. Electronic informed consent was obtained from the mothers before they completed the survey. To permit mothers to be fully informed before giving consent, we provided them with an information sheet on the study. The inclusion criteria were as follows: being a mother of a child from 5 to 10 years old, ability to read and understand Italian, and being a resident in Italy. The exclusion criteria were the presence of any pre-existing medical condition for the mother and for the child. At the end of the survey, a list of advice for the parent–child relationship during the lockdown was given. The Ethical Committee for Psychological Research of the Department of History, Society, and Human Studies (University of Salento) gave its approval for the research (No. 53162), confirmed by the Ethical Committee of the University of Bergamo as well.

### Participants

One hundred forty-four mothers participated (*M*_*age*_ = 39.3, 25–52, *SD* = 5.6) with children from 5 to 10 years old (*M*_*age*_ = 7.54, *SD* = 1.6; 82 boys). Of the questionnaires, 43% were completed for female children, and 57% were completed for male children. Of the families, 30.8% had only 1 child, 57.7% had 2 children, and 11.5% had more than 2 children. Of the mothers, 66% lived in Southern Italy, 14.8% lived in the North, and 14.1% lived in the center. The majority of the samples were employed (68.3%), 15.5% were unemployed, 14.1% were homeworkers, and 2.1% were students. Finally, the maternal educational level was clustered into three levels: low (8 years of education, corresponding to the Junior High School degree) for 7.7% of mothers, intermediate (13 years of education, corresponding to the High School degree) for 38% of them, and high (18 or more years of education, corresponding to a University degree) for 54.2% of the sample.

### Measures

#### Maternal Distress

The levels of distress were measured by applying the 21-item Depression Anxiety Stress Scale self-report measure [henceforth DASS-21; (43, 44)]. According to Bottesi et al. (44), in the current study, we conceived distress as a general trait characterized by a combination of depressive, anxious, and distress symptoms. The DASS-21 is a general measure of the individual's distress that required an evaluation considering the previous week corresponding in our case to a period of lockdown. Examples of items are “I tended to over-react to situations” (item 6), “I was worried about situations in which I might panic and make a fool of myself” (item 9), and “I was unable to become enthusiastic about anything” (item 16). Response options varied from 0 (“never happened”) to 3 (“always happened”). The final score was calculated as the mean of all the items, with higher scores indicating higher distress levels.

#### Maternal Coping

The 13-item Coping Scale [henceforth CS; (45)] was applied to measure general individual coping strategies. As Hamby et al. (45) suggested, maternal coping was defined as an integration of appraisal and behavioral methods of coping to deal with problematic situations. Specifically, the scale allows us to measure the appraisal (e.g., “When dealing with a problem, I try to see the positive side of the situation”) and the behavioral (e.g., “When dealing with a problem, I take steps to take better care of myself and my family for the future”) strategies applied by individuals to cope with a generic stressful situation. To mirror the distress evaluation described above, we required participants to refer to the previous week while they were under the lockdown condition. Response options ranged from 1 (“not true about me”) to 4 (“mostly true about me”). A total score was created as the mean of the scores of all the items, with higher scores indicating higher levels of coping.

#### Children's Emotional Responses

Due to the modality of recruitment, the emotional responses of the children to the lockdown were rated by their mothers. Seven mother-reported questions were created *ad hoc* for the present research (see Supplementary Materials), measuring her child's emotional responses during the previous week. Specifically, we considered a range of emotions and mental states (i.e., happy, sad, anxious, worried, angry, quiet, and secure). Response options ranged from 0 (“not at all”) to 4 (“very much”). Two scores were created: one for children's positive and one for children's negative emotions/mental states as the average of the scores of all items, with higher scores indicating higher experience of positive or negative emotions/state of mind.

#### Children's Adaptive Behaviors

This section includes four mother-reported questions created *ad hoc* for the present research (see Supplementary Materials), evaluating her child's adaptive behaviors during the previous 7 days. In particular, we asked mothers to say how much the child was involved in the following activities: playing as usual, talking about negative emotions, being involved in school activities, and spending free time independently/alone. Response options vary from 0 (“not at all”) to 4 (“very much”). We chose such outcomes, as these are aspects of a child's functioning that are more likely to be affected by a maladaptive response to the situation (46).

#### Exposure to COVID-19

This section of the questionnaire includes five items investigating individual exposure to COVID-19 infection (see Supplementary Materials). Specifically, we asked mothers if they, their partner, their relatives, and their friends were positive for the virus infection or manifested correlated symptoms. We also included a question asking whether someone among their relatives or friends died because of COVID-19. A cumulative score was obtained as the sum of items (*M* = 0.28; *d*s = 0.72; range 0–4), with higher scores indicating greater exposure. The exposed group included participants who had at least 1 point (*N* = 25); the non-exposed group included participants who reached 0 point (*N* = 119).

### Statistical Analysis

Preliminary statistical analyses were performed by SPSS version 25 (47), whereas the main analyses were conducted using the *Lavaan* and *SemPaths* packages (48) in RStudio software (49). There were no missing data. Mann–Whitney U-tests were performed to identify systematic differences in the distribution of distress and coping between individuals exposed or not exposed to COVID-19. Furthermore, Pearson's or Spearman's correlation coefficients were calculated for each variable in the model.

For the main analyses, the hypothesized model (Figure 1) was tested using structural equation modeling (SEM) considering the structural model. To address non-normality in the data, WLSMV was applied, and the Satorra–Bentler scaled-test statistic (50) and robust standard errors were used. In the model specification, the factor loading of the first indicator of each latent variable was set to 1. The following goodness-of-fit indices were used to evaluate model–data correspondence: the Chi-square-value, the Comparative Fit Index (CFI), and the Root Mean Square Error of Approximation (RMSEA). Given that the χ^2^ value is influenced by the sample size, it was considered, together with CFI and RMSEA. Byrne (2016) suggested accepting a model when the CFI is higher than 0.90 and close to 0.95 and when the RMSEA is 0.08 or less. Finally, modification indices and the matrix of standardized correlation residuals were inspected for potential improvement of model fit. Direct, indirect (or mediate), and total effects were also estimated. According to MacKinnon et al. (51), the direct effects reveal if a predictor (x) is related to an outcome (y) variable, the indirect effects show if the impact on the outcome variable (y) is explained through the intervening variable (*M*), and finally, the total effect includes both the direct and the indirect effects. The reversed model was tested following what was established for the main model. ΔChi-square was applied to compare the expected model and the reversed model.

## Results

### Preliminary Analyses

The factor structure of maternal distress, coping, and children's emotional reaction scales was calculated. For distress, the scale showed good internal consistency (α = 0.91, *r*s > 0.36). The confirmatory factor analysis (CFA) on the one-factor structure showed good fit of the data with a non-significant, χ^2^ (168) = 72.136, *p* > 0.05, CFI = 1, RMSEA = 0.03, CI90 (0.019, 0.044). The coping scale showed moderate internal consistency (α = 0.73, *r*s > 0.17), and the CFA showed a unidimensional factor structure with a significant, χ^2^ (65) = 176.831, *p* < 0.001, and from moderate to acceptable fit indices, CFI = 0.88, RMSEA = 0.07, CI90 (0.06, 0.09). Finally, the children's emotional reaction showed acceptable internal consistency (for the children's positive emotions: α = 0.74, *r*s > 0.36; for the children's negative emotions: α = 0.77, *r*s > 0.44; for the total scale: α = 0.66, *r*s > 0.32). The CFA showed a good fit of the data with a significant, χ^2^ (13) = 42, *p* < 0.001, CFI = 0.95, RMSEA = 0.08, CI90 (0.06, 0.12).

Mann–Whitney U-tests revealed significant differences in maternal distress and coping levels, with mothers exposed to COVID-19 reaching higher means than those not exposed. Table 1 shows the means and standard deviations of the two groups.

**Table 1:** Mean scores, standard deviations, and results of the non-parametric *t*-tests.

	**Exposed to COVID-19 (*N* = 119) *M* (*SD*)**	**Non-exposed to COVID-19 (*N* = 25) *M* (*SD*)**	**Mann–Whitney U-test**
Distress	1.6 (0.37)	1.5 (0.37)	*U* = 8,099.5*
Coping abilities	2.7 (0.40)	2.5 (0.47)	U = 8,111*

* *p < 0.05*.

Table 2 shows the correlations between measures. The distress of the mothers and their coping abilities correlated with children's positive and negative emotions. The distress of the mothers correlated with children's playing activities, school-related activities, and time spent alone. Mothers' coping abilities correlated with children's playing activities, school-related activities, and time spent alone. Children's positive emotions correlated with playing activities, school-related activities, and time spent alone. Children's negative emotions correlated with playing activities, school-related activities, time spent alone, and tendency to talk about negative emotions. Children's positive and negative emotions were correlated.

**Table 2:** Correlation results between variables.

	**1**	**2**	**3**	**4**	**5**	**6**	**7**
Maternal distress	−0.315***	−0.342***	0.443***	−0.353***	−0.307***	−0.311***	0.105
Maternal coping abilities (1)		0.212**	−0.209*	0.257**	0.205*	0.287***	0.161
Children's positive emotions (2)			−0.598**	0.470**	0.350***	0.430***	−0.114
Children's negative emotions (3)				−0.342**	−0.332***	−0.377***	0.343***
Children's playing activities (4)					0.330***	0.462***	0.046
Children's school-related activities (5)						0.531***	−0.079
Children's time spent alone (6)							0.010
Children's tendency to talk about negative emotions (7)							

*p < 0.05; **p < 0.010; and ***p < 0.001.

### Main Model

**Figure 2:**
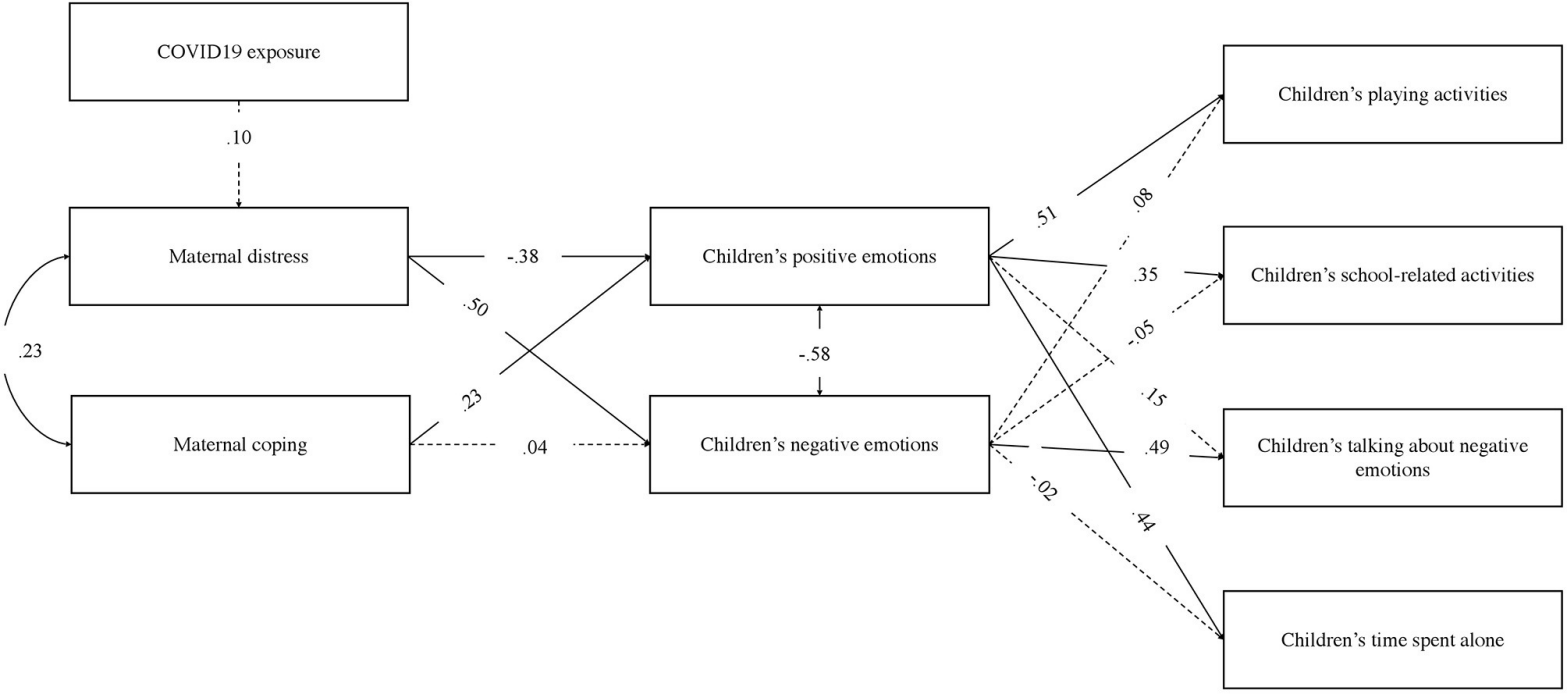
The final model. Only standardized path coefficients are shown. Dashed arrows denote non-significant paths; all other paths denote significant relationships at *p* < 0.05.

The hypothesized model in Figure 1 was tested with COVID-19 exposure as a covariate. The model reached a good fit of the data, χ^2^ (15) = 26.44, *p* = 0.03, CFI = 0.98, RMSEA = 0.05, 90% CI90 (0.014, 0.084). Figure 2 shows the path coefficients.

COVID-19 exposure was not significantly related to distress of the mothers. Distress and coping correlated (β = 0.23, *p* = 0.001) with children's positive and negative emotions as well (β = −0.58, *p* < 0.001). Distress was significantly negatively related to the children's positive emotions (β = −0.38, *p* < 0.001) and positively related to the children's negative emotions (β = 0.50, *p* < 0.001), indicating that increased levels of distress were associated with higher children's negative emotions and lower children's positive emotions, as reported by mothers. Coping was significantly related to children's positive emotions (β = 0.23, *p* < 0.001) but not to their negative emotions. Children's positive emotions were significantly related to play activities (β = 0.51, *p* < 0.001), school-related activities (β = 0.35, *p* < 0.001), and time spent alone (β = 0.44, *p* < 0.001). Children's negative emotions were significantly related to talking about negative emotions (β = 0.49, *p* < 0.001).

Direct, indirect (or mediate), and total effects of maternal distress and coping abilities (predictive variables) on children's behaviors (outcome variables) are reported in Table 3, together with the kappa-squared values. All the estimated indirect effects were significant, meaning that maternal distress and maternal coping were associated with children's behaviors through the mediation effects of children's positive and negative emotions. Total effects were all significant as well. There were also four direct effects between maternal distress, children's playing activities, and time spent alone and between maternal coping, children's school-related activities, and time spent alone.

**Table 3:** Path analysis of mediation effects applying SEM.

	**Direct effect**	**Indirect effect**	**Total effect**	***k*^**2**^**
**Path: maternal distress** **→** **children's positive emotions** **→** **children's outcomes**				
Children's playing activities	β = −0.322***	β = −0.257*	β = −0.579***	0.44
Children's school-related activities	β = −0.150	β = −0.284***	β = −0.434**	0.65
Children's time spent alone	β = −0.306*	β = −0.276***	β = 0.581***	0.47
**Path: maternal distress** **→** **children's negative emotions** **→** **children's outcomes**				
Children's tendency to talk about negative emotions	β = 0.059	β = 0.361***	β = 0.419***	0.86
**Path: maternal coping abilities** **→** **children's positive emotions** **→** **children's outcomes**				
Children's playing activities	β = 0.108	β = 0.145**	β = 0.253*	0.57
Children's school-related activities	β = 0.295**	β = 0.116**	β = 0.411***	0.28
Children's time spent alone	β = 0.437***	β = 0.116**	β = 0.553***	0.21

*p < 0.05; **p < 0.010; ***p < 0.001; and k^2^ = mediator effect size.

Significant correlations were found between the age of the mothers and distress (*r* = −0.26, *p* = 0.002) and the age of the children and distress (*r* = −0.179, *p* = 0.031). We then tested the same model adding the age of the mothers and children as covariates. The model reached a good fit of the data, χ^2^ (29) = 34.081, *p* > 0.05, CFI = 0.98, RMSEA = 0.03. Nevertheless, mother's age was not significantly related to distress, whereas children's age was significant but with a low beta (β = −0.039, *p* = 0.03).

Since the design of the study was cross-sectional, the reverse model was tested, reaching a worse fit of the data, χ^2^ (21) = 220.507, *p* < 0.001, CFI = 0.65, RMSEA = 0.16, 90% CI90 (0.16, 0.21). This demonstrated that the expected model was the best fit of the data compared with the reverse one.

## Discussion

The spread of COVID-19 emergence, together with the non-pharmacological health measures adopted to reduce contagion (i.e., lockdown and physical isolation), challenged researchers to rapidly understand the impact of the situation on people's mental health. The present research intends to contribute in this debate by focusing on the relations between maternal distress/coping and children's behaviors during the COVID-19 lockdown, hypothesizing a mediation effect *via* children's emotional experience. Overall, our results show that in the challenging time of the lockdown, mothers' level of experienced distress and their ability to cope with it played a role in the level of adjustment displayed by their children *via* both direct and indirect influences. Maternal stress/coping can directly affect the child's adaptive behaviors, as the child might be led to modify his/her behaviors in response to maternal difficulties/resources (without being emotionally affected or only in part). Maternal stress and coping can also indirectly affect the child's adaptive behaviors, as maternal emotions might influence the child's emotions and, in turn, the child's emotions determine the child's behaviors. In the following sections, we first address differences in the levels of distress and coping shown by mothers in our sample, then we turn to our key findings, and we close with reflections on the limitations of our study and on the applicative suggestions coming from our results. To the best of our knowledge, our data are among the first to provide a picture of how lockdown affects families in terms of the interplay between parenting and children's outcomes.

### Maternal Distress/Coping Skills During the Lockdown

As expected, mothers with higher exposure to COVID-19 showed higher levels of distress and higher displays of coping attitudes and behaviors to try to face difficult situations. These psychological efforts to promptly react and adapt to COVID-19 emergence, when protracted in time, could be responsible for the mental health issues reported in previous studies (8–12). However, the results of our SEM analyses showed that COVID-19 exposure was not a predictor of mothers' distress. These results deserve attention and permit some constructive considerations on the factors contributing to distress in the family during COVID-19 emergence. First, we have to consider the features of our sample. The majority of our participants were from Southern Italy, an area not heavily affected by the virus (52), with the consequence that the variance in the levels of exposure displayed by our sample was rather small. For future research, it will be interesting to see if such results would change in areas or regions where the rates of COVID-19 affections have been higher or in a sample exposed to COVID-19. If the exposure to COVID-19 was limited in our sample, the same cannot be claimed for the exposure to social distance measures. The Italian government ordered a pre-cautionary lockdown in all the national territories, regardless of their rates of affections. Our results are consistent with international literature (53), suggesting that high uncertainty situations (due, for example, to incomplete and inconsistent information, uncertainty over disease status, and the duration of the lockdown itself) represent a risk factor for the development of negative psychological mental health. The spread of COVID-19 indeed put people in direct contact with high levels of uncertainty due to the possibility of being infected and of facing serious medical complications, together with the uncertainty for future life.

### Interplay Between Mothers' Distress/Coping Strategies and Children's Emotional/Behavioral Adjustment

Overall, our data suggested that distress represented a risk factor for children's adaptive behaviors during an uncertainty situation, such as the one participants experienced under the COVID-19 lockdown, as distress might buffer the maternal recognition of children's positive emotions and, at the same time, might trigger the maternal recognition of children's negative emotions. On the other hand, coping skills represented a protective factor, but they exerted their action preferentially on the recognition of children's positive emotions.

Our findings confirmed our initial expectations that maternal distress during the COVID-19 lockdown would have had an effect on children's adaptive behaviors through both a direct effect and the mediation of children's negative and positive emotional reactions (Hypothesis 1). Specifically, 65.4% of the overall effect from maternal distress to the child's involvement in school-related activities is explained by the mediating effect of the child's positive emotions as reported by mothers. Furthermore, 86.1% of the overall effect of maternal distress on children's tendency to talk about negative emotions is explained by children's negative emotions acting as mediators of the effect. These results are in line with the literature showing that greater parental distress significantly predicts greater children's anger, anxiety, and withdrawal (54, 55), and that conversely, positive parenting elicits children's positive emotions (56). More than one possible explanation can make sense of this relation. Following the model of Contagion of Affect Induction by Denham (57), a negative affective environment may sensitize children and make them vulnerable to negative feelings, and a less distressed affective context may make the child feel safe, thus inducing more children's positive emotions (27, 58). Moreover, parental distress influences parenting behaviors (20, 59), such as parents report high levels of distress risk to exhibit scarce warmth and responsiveness when interacting with their children, to adopt inconsistent discipline, and to have expectations for their children that are unaligned with their capacities (20, 60, 61). These parental behaviors and attitudes, in turn, are detrimental to the adjustment of the child in terms of emotions and behaviors (59, 62).

The pattern of results in the present study also corroborated with Hypothesis 2 on how maternal coping abilities during the COVID-19 lockdown might have affected children's adaptive behaviors, but through the mediation of children's positive emotional reactions only. Specifically, we found that 57.3% of the overall effect of maternal coping abilities on children's involvement in playing activities can be explained by the mediating effect of the child's positive emotions. The mediation path was not significant for children's negative emotions. Our findings are aligned with theoretical models positing that parents who are able to deal with distress are in conditions to provide a consistent caregiving environment, even in difficult circumstances, that in turn promotes positive feelings in children (63). The literature is consistent in indicating that good coping abilities facilitate the adoption of adaptive parenting behaviors (64), and that the coping style of the parent is associated with the coping style of the child *via* the mechanisms described by the Social Learning Approach (26, 65). In particular, there are three classes of positive coping mechanisms that have been found to be beneficial in highly stressful situations (66): positive reappraisal, problem-focused coping, and the creation of positive events.

Parental distress is characterized by multiple factors (20, 67, 68). In line with this, during the lockdown, mothers were exposed to a certain amount of distress that everybody (not only parents) experienced, such as social isolation, but also to a certain amount of distress specifically linked to their parental role, such as worrying about their own children's well-being or school experience. Moreover, for some families, the levels of experienced distress were even higher due to critical conditions, such as economic issues, marital problems, and taking care of fragile relatives, such as disabled children or elderly parents. It is also worth noting that during the lockdown, job conditions (e.g., health-care professionals and occasional workers) might have influenced the levels of distress. Future research should investigate the unique roles of each of these stressing factors in the interplay between parental and children's adjustment during a lockdown situation. Similarly, transactional models of development (69, 70) consider reciprocal influences between the parent and the child so that parenting distress is known to play a role in the child's behavioral adjustment, but at the same time, a high level of child behavioral problems is responsible for an increase in parenting distress (71, 72). Given this, future research on quarantine and lockdown situations should better understand the overall pattern of the reciprocal effects between children's and parents' responses to the crisis.

### Limitations

Our study presents some limitations as well. First, we measured children's emotional reactions and behaviors enquiring their mothers. Although the evaluation may be biased by maternal psychological functioning, such as worries or tiredness, the lockdown conditions impeded the possibility of measuring children's emotional reactions involving children directly. Future studies may be interested in administering children-reported measures applicable during a lockdown period, such as online story-telling or drawings, to assess their mental states and adaptive behaviors during a prolonged lockdown situation. The second limitation pertains to the fact that the measures of coping and children's emotional and behavioral functioning are not validated in the context of Italy. Third, the study was cross-sectional with a small sample size, and no considerations can be drawn regarding causality. Furthermore, the recruitment process through social media might have created a bias in the sample composition, which may have reduced the generalizability of our results. We also should notice that the mothers for the most part came from the South of Italy, a place in Italy in which the spread of the pandemic was less serious. This could have affected the results even if the population in the South has lived the same level of emergency and uncertainty of the population in the North. Finally, our data included only mothers, and we collected less information on their socio-demographic data. Future studies might be interested in investigating the same patterns of relationships between variables considering the father's point of view and the role of possible mediators and moderators that our design did not include. Good candidates are the actual parenting behaviors, the psychological characteristics of the child, such as mentalization and trust (73–77), and the distribution of the caregiving responsibilities among family members.

### Conclusions

The present study brings a contribution regarding the interplay between mother's functioning and child's functioning during the lockdown due to COVID-19. The research findings highlighted that distress represented a risk factor for children's adaptive behaviors during an uncertainty situation, buffering maternal recognition of children's positive emotions and, at the same time, triggering the maternal recognition of children's negative emotions. On the other hand, coping skills represented a protective factor, exerting their action preferentially on the recognition of children's positive emotions

Findings of this study suggest the following recommendations for policy and interventions. More attention needs to be paid to vulnerable groups, such as mothers and children, and as a follow-up of this point, a nationwide planning for first aid should be implemented, in which parents receive support and adequate information to manage the relationship with their children. Targeted interventions should be built to reduce psychological distress, focusing on risk and protective factors [see also (78, 79)]. Communication during health crisis tailored on families' needs should be developed, even considering building trust in officials (80) and health authorities (81) to enhance the probability that the population follows the non-pharmaceutical established procedures and cope with them. Finally, children should be the target of the communication as well as their parents to reduce even their psychological burden [see (82)] and prevent future mental health problems.

## Data Availability Statement

The raw data supporting the conclusions of this article will be made available by the authors, without undue reservation.

## Ethics Statement

The studies involving human participants were reviewed and approved by Department of History, Society, and Human Studies, University of Salento, Via di Valesio, 73100 Lecce, Italy. The patients/participants provided their written informed consent to participate in this study.

## Author Contributions

SP, FL, and AL conceptualized the study. AL and SP developed the survey on Qualtrics and analyzed the data. SP, FB, and AL wrote the draft paper. IC and FL revised the manuscript and provided feedback. All authors recruited the participants, read, and approved the final version of the manuscript.

## Conflict of Interest

The authors declare that the research was conducted in the absence of any commercial or financial relationships that could be construed as a potential conflict of interest.
