# Supplementary material for: Maternal Distress/Coping and Children's Adaptive Behaviors During the COVID-19 Lockdown: Mediation Through Children's Emotional Experience
Source: Front Public Health. 2020 Nov 19;8:587833. doi: 10.3389/fpubh.2020.587833 (PMC7711130; doi:10.3389/fpubh.2020.587833)
Supplement: Supplementary file 1 [file Table_1.docx]

**Maternal distress/coping and children’s adaptive behaviors during the COVID-19 lockdown: Mediation through children’s emotional experience**

**Petrocchi, S., Levante, A., Bianco, F., Castelli, I., & Lecciso, F.**

***Supplementary Material***

The present supplementary material contains the questions asked to parents regarding their children’s emotional responses and adaptive behaviors (see points 1 and 2), and questions on COVID-19 exposure (see point 3). Moreover, we included a final section (see point 4) with the advice we gave to the parents at the end of the survey.

# Children’s emotional responses

# Please, read the following questions regarding your child's emotional responses. Please, mark the answer indicating how much your child felt the emotion during the previous week.

|  | 0  Not it all | 1  A few | 2  Quite | 3  Much | 4  Very much |
| --- | --- | --- | --- | --- | --- |
| During the previous week, how much do you think your child was HAPPY?^*^ | 0 | 1 | 2 | 3 | 4 |
| During the previous week, how much do you think your child was SAD? ^**^ | 0 | 1 | 2 | 3 | 4 |
| During the previous week, how much do you think your child was ANXIOUS? ^**^ | 0 | 1 | 2 | 3 | 4 |
| During the previous week, how much do you think your child was WORRIED? ^**^ | 0 | 1 | 2 | 3 | 4 |
| During the previous week, how much do you think your child was ANGRY? ^**^ | 0 | 1 | 2 | 3 | 4 |
| During the previous week, how much do you think your child was QUIET? ^*^ | 0 | 1 | 2 | 3 | 4 |
| During the previous week, how much do you think your child was SECURE? ^*^ | 0 | 1 | 2 | 3 | 4 |

# Note: ^*^ = indicates positive emotions/states of mind; ^**^ = indicates negative emotions/states of mind.

# 2. Children’s adaptive behaviors

# Please, read the following questions regarding your child's adaptive behaviors. Please, mark the answer indicating how much your child expressed the following behaviors during the previous week.

|  | 0  Not it all | 1  A few | 2  Quite | 3  Much | 4  Very much |
| --- | --- | --- | --- | --- | --- |
| During the previous week, how much did your child play as usual? | 0 | 1 | 2 | 3 | 4 |
| During the previous week, how much did your child talk about negative emotions? | 0 | 1 | 2 | 3 | 4 |
| During the previous week, how much was your child involve in school-related activities? | 0 | 1 | 2 | 3 | 4 |
| During the previous week, how much did your child spend free time independently/alone? | 0 | 1 | 2 | 3 | 4 |

# COVID-19 exposure

Please read the following questions regarding the exposure to COVID-19. Mark the answer indicating the most suitable answer for you.

1. Have you ever tested positive to COVID-19?
   - 1. Yes
     2. No
     3. I had related symptoms, but I have not being tested
2. Has your partner tested positive to COVID-19?
   - 1. Yes
     2. No
     3. He/She had related symptoms, but he/she has not being tested
     4. I do not have a partner
3. Has someone from your family tested positive to COVID-19?
   - 1. Yes
     2. No
     3. Someone had related symptoms, but they have not being tested
     4. I do not know
4. Has someone from your friends tested positive to COVID-19?
   - 1. Yes
     2. No
     3. Someone had related symptoms, but they have not being tested
     4. I do not know
5. Did some of your family members or friends die because of COVID-19?
   - 1. Yes
     2. No
     3. I do not know

For items 1 to 4, response options “1” and “3” have being scored as 1. Response options “2” and “4” have being scored as 0. For the fifth item, response option “yes” was scored as 1 and “no” or “I do not know” as 0.

**4. Final advices for parents**

The following text has been implemented on the survey to be showed to mothers at the end of the compiling.

Below, you may find some advice, for you and for your child, developed by the research team according to the Italian National Psychology Association guidelines. We hope they may help you and your child during the emergency due to COVID-19.

1. Anxiety and worries during an emergency, such as COVID-19 pandemic, are natural and appropriate emotions. We are now experiencing a complicated situation, and the right dose of anxiety might help us to display appropriate behaviors to protect us from danger.

2. Each of us has the resources to deal with the situation.

3. We should maintain stable routines, such as wake up, having meals, dedicate time for work- or school-related activities at the same time.

4. Sharing anxiety and fear with someone is a great way to deal with what we consider as scary.

5. We should try to spend a part of our day doing something not related to the COVID-19, such as listening to music, watching movies, reading books. Or you may want to be involved in those activities you have postponed for lack of time (gardening, light housework, take a diary)

6. Remember that parental stress influences children, although they may express stress differently from the adult. For example, children may appear to be less interested in their favorite activities or, on the contrary, more active than usual.

8. Try to dedicate a special moment with your child, playing with him/her, reading a book together, or sing a song.

9. If you want to talk to your child about the situation, try to use simple and understandable words. Do not hide him/her the truth, but explain that the situation will last for a limited time. Finally, try to highlight the positive aspects of the situation we are experiencing (e.g., more time with mom).

10. As a routine, before going to bed, try to relax with your child without thinking and talking about COVID-19: the night rest is essential for our well-being.
